# Interactions of occult tumor spread and surgical technique on overall and disease-free survival in patients operated for stage I and II right-sided colon cancer

**DOI:** 10.1007/s00432-021-03773-6

**Published:** 2021-08-24

**Authors:** G. S. Banipal, B. V. Stimec, S. N. Andersen, A. E. Faerden, B. Edwin, J. Baral, J. Šaltytė Benth, D. Ignjatovic, The RCC study group, Tom Oresland, Arne O Bakka, Yngve Thorsen, Anne Negaard, Jens Marius Nesgaard, Russel Jacobsen, Kari Mette Langerød von Brandis, Tania Hansen, Pål Suhrke, Javier Armando Luzon, Barış Sevinç, Bjarte Tidemann Andersen, Roberto Bergamaschi, Ulrich Schneider, Marcos Gomez Ruiz, Erik Kjaestad, Vahid Bemanian, Jacob Ghotbi, Anne Pernille H. Dyrbekk

**Affiliations:** 1 Department of Digestive Surgery, Akershus University Hospital HF, Postboks 1000, 1478 Lorenskog, Norway; 2 Anatomy Sector, Teaching Unit, Faculty of Medicine, University of Geneva, Geneva, Switzerland; 3 Department of Pathology, Akershus University Hospital HF, Postboks 1000, 1478 Lorenskog, Norway; 4 Interventional Centre and Department of HPB Surgery, Rikshospitalet, Oslo University Hospital, Oslo, Norway; 5 Department of Colorectal Surgery, Klinikum Karlsruhe, Karlsruhe, Germany; 6 Institute of Clinical Medicine, Faculty of Medicine, University of Oslo, Oslo, Norway; 7 Health Services Research Unit, Akershus University Hospital HF, Postboks 1000, 1478 Lorenskog, Norway

**Keywords:** Stage I/II right-side colon cancer, Micro metastases, Isolated tumor cells, D3 right colectomy, Occult tumor spread

## Abstract

**Purpose:**

To determine if “medial to lateral” (ML) dissection with devascularization first is superior to “lateral to medial” (LM) dissection regarding numbers of lymph node micro metastases (MM) and isolated tumor cells (ITC) as well as 5-year disease-free (5YDFS) and 5-year overall survival (5YOS) in stage I/II right-sided colon cancer.

**Methods:**

Two datasets are used. ML group consists of consecutive stage I/II patients from a prospective trial. LM group is the original dataset from a previous publication. All harvested lymph nodes are examined with monoclonal antibody CAM 5.2 (immunohistochemically). Lymph node harvest and 5YOS/5YDFS were compared between ML/LM groups, stage I/II tumors and MM/ITC presence/absence.

**Results:**

117 patients included ML:51, LM:66. MM/ITC positive in ML 37.3% (19/51), LM 31.8% (21/66) *p* = 0.54. The 5YDFS for patients in ML 70.6% and LM 69.7%, *p* = 0.99, 5YOS: 74.5% ML and 71.2% LM (*p* = 0.73). No difference in 5YDFS/5YOS between groups for Stage I/II tumors; however, LM group had an excess of early tumors (16) when compared to ML group, while lymph node harvest was significantly higher in ML group (*p* < 0.01) 15.1 vs 26.7. 5YDFS and 5YOS stratified by MM/ITC presence/absence was 67.5%/71.4%, *p* = 0.63, and 75.0%/71.4%, *p* = 0.72, respectively. Death due to recurrence in MM/ITC positive was significantly higher than MM/ITC negative (*p* = 0.012).

**Conclusion:**

Surgical technique does not influence numbers of MM/ITC or 5YDFS/5YOS. Presence of MM/ITC does not affect 5YOS/5YDFS but can be a potential prognostic factor for death due to recurrence.

**Clinical trial:**

Safe Radical D3 Right Hemicolectomy for Cancer through Preoperative Biphasic Multi-Detector Computed Tomography (MDCT) Angiography” registered at http://clinicaltrials.gov/ct2/show/NCT01351714 .

## Introduction

Starting in the mid-eighties, a rise in interest for micro metastases (MM) and isolated tumor cells (ITC) in patients with stage I/II colon cancer was noted. The main focus of these articles was mostly their prognostic value, whereas surgical technique was seldom addressed. Up to date the controversy of the prognostic value of MM/ITC has not been resolved. Some articles report poorer long-term survival and higher recurrence rates (Faerden et al. 2011; Schaik et al. 2009 May; Sloothaak 2017; Weixler 2016), while others do not (Kronberg et al. 2004; Hong et al. 2017). Data from the literature imply that MM and ITC occur in regional lymph nodes in 4.2–41% (Schaik et al. 2009; Sloothaak 2017) and 19–31% (Sloothaak 2017; Weixler 2016) of patients, respectively. The underlying reason for this high variability can lie in the methodology used to verify the cells but can also be dependent on the specimen mobilization technique deployed at surgery. Furthermore, studies, where results are stratified according to colon segments are scarce, when known that lymph node numbers for different colon segments can differ significantly (Trepanier et al. 2019; Hohenberger et al. 2009; Malik et al. 2020). Unfortunately, most publications include the right, left colon as well as rectum and fail to report the mode of access or specimen mobilization technique.

The impact of specimen mobilization technique on cancer cell migration (medial/lateral (ML) vs. lateral/medial (LM)) has been debated since 1967(Turnbull et al. 1968). When MM/ITC are concerned the literature contains few studies. Among these a study that compared numbers of MM/ITC between patients operated with laparoscopy to those operated with laparotomy after all patients underwent a sentinel node procedure, demonstrated only significantly higher numbers of ITC in the laparotomy group (Zaag 2011).

This study aims to compare the effect of specimen mobilization technique on MM/ITC numbers in the surgical specimen, as well as on long-term survival in patients operated for stage I/II right-sided colon cancer. A second aim is to establish the overall prognostic value of MM/ITC on 5YDFS and 5YOS.

## Material and methods

### Two sources of data were used for comparison.

#### ML group

The first dataset was obtained from the ongoing prospective multicenter study entitled “Safe Radical D3 Right Hemicolectomy for Cancer through Preoperative Biphasic Multi-Detector Computed Tomography (MDCT) Angiography” registered at http://clinicaltrials.gov/ ct2/show/NCT01351714 and ethically approved by Regional ethical committee, South-East Norway (REK Sør-Øst) no. 2010/3354. Patients older than 18 years of age with potentially curable colon cancer were included after written consent. The surgical specimens from patients operated at Akershus University Hospital (AHUS) and the Vestfold Hospital Trust (SIV) from October 2011 to February 2014 and Viszeralchirurgie Klinikum Karlsruhe, Germany (KR) from 2017 to 2018 were analyzed at the respective departments of pathology.

Patients were operated through laparotomy when a medial to lateral (devascularization first) approach was deployed (ML). The surgical specimen (Spasojevic et al. 2013; Nesgaard et al. 2015) was divided into the respective level of dissection (D2 and D3) volumes after specimen removal through a line 10 mm towards the right of the superior mesenteric vein beginning at 10 mm caudal to the ileocolic artery origin and ending at 5 mm cranial to the middle colic artery origin as specified in project protocol. Both resection specimens (D2/D3) were preserved and sent to routine pathological examinations separately. Only the D2 volume was used for further analysis to make the surgical specimens comparable between the two groups 5 patients were excluded due to 30 days mortality and stage III patients.

#### LM group

The second dataset used for comparison was a subgroup of patients compiled from the raw data used in a previous publication (Faerden et al. 2011), where D2 lymph node dissection was the standard of surgery. This subgroup consisted of both right and left colon and sigmoideum cancer patients, where seven patients with 30-day mortality were excluded. Further exclusions were patients not suffering from right-sided colon cancer as well as all patients with stage III disease. The remaining dataset contained only patients operated for right-sided stage I/II colon cancer, all through lateral to medial access (LM) in contrast to the previously described trial. Regional ethical approval and signed informed consent for these patients was documented in the article.

#### Histopathology

The same pathology laboratory and pathologist performed the analyses in this study as in the current patient series Solveig Norheim Andersen (SNA) in Ahus, while Ulrich Schneider (US) performed the analyses in KR.

All lymph nodes within the D1&2 volume were examined by routine microscopy, i.e., 3- to 4- µm hematoxylin and eosin (H&E) stained sections. Lymph nodes larger than 3 mm in diameter were divided into two or more parts parallel to the longest axis. Further, the nodes were examined immunohistochemically using the CAM 5.2 monoclonal antibody (Becton Dickson, Mountain View, CA, USA). CAM 5.2 was chosen due to the distinct cytoplasmic staining of epithelial cells and very little unspecific staining. These nodes were examined as follows: two 3- to 4- µm thick sections, separated by approximately 200 µm, were cut from different levels and mounted on coated slides. After antigen retrieval by microwaving (20 min at 100 °C), immunostaining was performed in an Autostainer (Dako Corporation, Carpentina, USA), using the monoclonal antibody CAM5.2. The Envision System was used for enhancing the signal with diaminobenzidine (DAB) as chromogen and Hagen’s hematoxylin for counterstaining and visualization of tissue structures. All immunohistochemically stained lymph node sections were examined by the same pathologist in two hospitals, while the samples from KR were analyzed by US. Only cells with distinct and deep cytoplasmic staining and atypical nucleus lying in the sinus system of the lymph nodes were counted.

Detection of metastasis both in clusters of cells or individual cells were graded in accordance with tumor node metastasis (TNM) staging system of the American Joint Committee on Cancer (AJCC) (Brierley and Wittekind 2017) as follows: Malignant cell cluster larger than 2 mm in diameter as ordinary metastasis, Malignant cell cluster between 0.2 and 2 mm as micrometastasis (MM), Malignant cell cluster less than 0.2 mm in diameter or single isolated tumor cell as isolated tumor cells (ITC).

#### Adjuvant treatment

Patients diagnosed with stage I/II were not offered adjuvant treatment, as directed by the Norwegian Guidelines for Colorectal cancer. None of the patients in stage I/II with positive MM/ITC received adjuvant treatment. Patients with stage III disease were excluded from further analysis.

#### Regrouping of patients

After calculating the results, the patients from groups ML and LM were regrouped into MM/ITC positive (MM/ITC +) and MM/ITC negative (MM/ITC −) and reanalyzed.

#### Statistical analysis

Data were described as frequencies and percentages or means and standard deviations (SD), as appropriate. Groups were compared by *χ*^2^ test for categorical and independent samples *t* test for continuous variables. Kaplan –Meier curves and log-rank test were used to compare 5YDFS and 5YOS between LM and ML, and between MM/ITC + and MM/ITC − groups. Cox regression analyses were used to adjust the differences in 5YDFS and 5YOS for demographic and clinical characteristics (gender, age, lymph modes number, tumor differentiation, Stage I/II tumor). The results with a P-value less than 0.05 were considered statistically significant. The statistical Product and Service Solutions Software (SPSS, Inc. Chicago IL) version 27 for windows was used for statistical analyses.

## Results

**Fig. 1:**
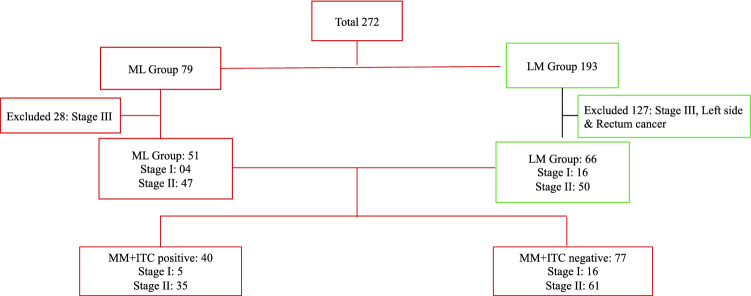
Demographics of two comparative groups. *LM* Lateral to medial dissection; *ML* Medial to lateral dissection; MM micro metastases; ITC Isolated tumor cells

A total of 272 patients were collected from the two data sources (79 ML + 193 LM). After exclusions (stage III disease, cancer locations not in the right colon) 117 patients remained eligible for the study, leaving 51 in the ML and 66 in the LM group (Fig. 1). Demographics and clinical characteristics for these two groups are presented in Table 1. The groups were comparable for sex, age and T stadium, while the LM group contained significantly more Stage I patients when compared to the ML (*p* = 0.012). The ML group contained significantly more low differentiated tumors (*p* = 0.005) and a higher lymph node harvest (*p* < 0.001).

**Table 1:** Demographics in ML and LM Groups

Parameters	ML *N* = 51	LM *N* = 66	*p* value
Sex			
Male, *n* (%)	19 (37.3)	27 (40.9)	0.688*
Female, *n* (%)	32 (62.7)	39 (59.1)	
Age (years)			
Total, mean (SD)	66.6 (9.6)	69.4 (12.6)	0.165**
Male, mean (SD)	65.4 (9.9)	69.5 (12.1)	0.231**
Female, mean (SD)	67.3 (9.5)	69.4 (13.1)	0.431**
Stage			
I, *n* (%)	5 (9.8)	19 (28.8)	0.012*
II, *n* (%)	46 (90.2)	47 (71.2)	
Tumor Differentiation			
Low, *n* (%)	6 (11.8)	3 (4.5)	0.005*
Moderate, *n* (%)	33 (64.7)	59 (89.4)	
High, *n* (%)	12 (23.5)	4 (6.1)	
TNM stage; T			
T1, *n* (%)	0	3 (4.5)	0.030*
T2, *n* (%)	5 (9.8)	16 (24.2)	
T3, *n* (%)	42 (82.4)	46 (69.7)	
T4, *n* (%)	4 (7.8)	1 (1.5)	
Number of D2 lymph *n*odes, mean (SD)	26.7 (14.7)	15.1 (7.1)	< 0.001**
Micrometastases			
*n*o, *n* (%)	32 (62.7)	45 (68.2)	0.539*
MM + ITC, *n* (%)	19 (37.3)	21 (31.8)	
MM/ITC by Stage			
ITC			
Stage I, *n* (%)	1/5 (20.0)	5/19 (26.3)	
Stage II, *n* (%)	17/46 (37.0)	3/47 (6.4)	
MM			
Stage I, *n* (%)	0/5	0/19	
Stage II, *n* (%)	1/46 (2.2)	3/47 (6.4)	
Long-term survival independent of MM/ITC			
Overall survival, *n* (%)	38 (74.5)	47 (71.2)	0.692*
5-year disease-free survival	36 (70.6)	46 (69.7)	0.917*
Long-term survival independent of MM/ITC by Stage I/II			
Stage I			
Overall survival, *n* (%)	5/5 (100)	13/19 (68.4)	0.147*
5-year disease-free survival, *n* (%)	5/5 (100)	13/19 (68.4)	0.147*
Stage II			
Overall survival, *n* (%)	33/46 (71.7)	34/47 (72.3)	0.948*
5-year disease-free survival, *n* (%)	31/46 (67.4)	33/47 (70.2)	0.769*
Death due to recurrence, *n* (%)	2 (3.9)	6 (9.1)	0.272*

*LM* Lateral to medial dissection, *ML* Medial to lateral dissection, *MM* micro metastases, *ITC* isolated tumor cells

*χ^2^ test

**Independent samples *t* test

**Fig. 2:**
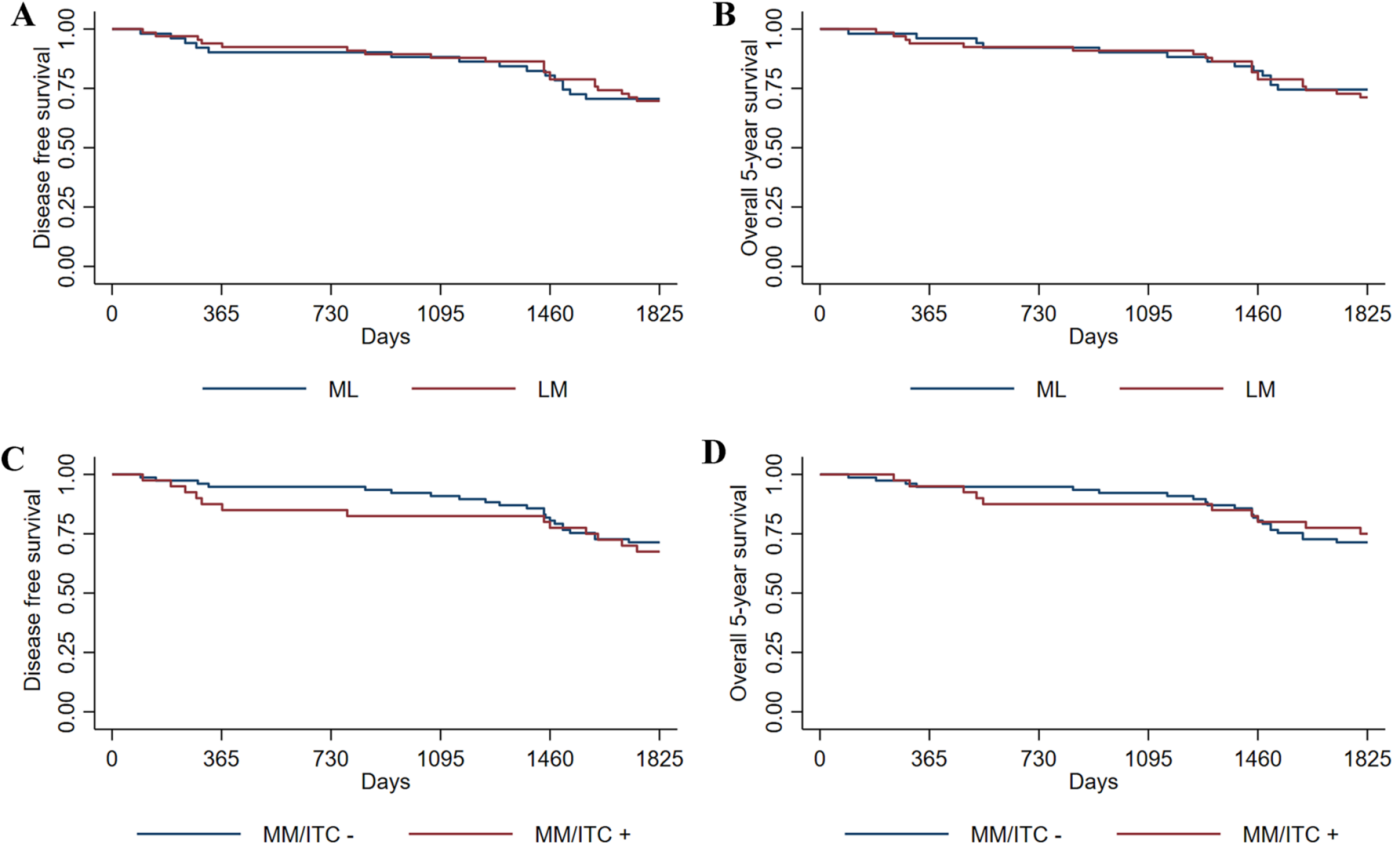
Kaplan–Meier 5YDFS and 5YOS in ML group vs LM group (**A**, **B**). Kaplan–Meier Disease 5YDFS and 5YOS in MM/ITC positive and negative group (**C**, **D**). *LM* Lateral to medial dissection; *ML* Medial to lateral dissection. *MM*/*ITC* +  micro metastases/Isolated tumor cells positive. *MM*/*ITC* − micro metastases/isolated tumor cells negative

Overall results showed no significant differences in numbers of MM/ITC (*p* = 0.54) between the groups (Table 1). Further analysis showed no statistically significant differences in 5YOS (*p* = 0.73) and 5YDFS (*p* = 0.99) (Fig. 2A and Fig. 2B) according to a long-rank test. In the LM group 6 patients (9.1%) died from recurrence, and 2 patients (3.9%) in the ML group (*p* = 0.27). Stratified by disease stage (stage I/II), no significant differences were found between the groups in 5YOS (*p* = 0.15/0.15) and 5YDFS (*p* = 0.95/0.77), respectively (Table 1).

When the data were stratified according to the presence of MM/ITC (Fig. 1) there were 40 patients in the MM/ITC + and 77 patients in the MM/ITC −. The groups were comparable for sex, age, stage of disease, tumor differentiation, tumor (T) stage and the mean number of harvested lymph nodes (Table 2). The distribution of MM/ITC in the whole group and stratified by the disease stage is presented in Table 2. There was no significant difference in 5YOS (*p* = 0.72), 75.0% vs. 71.4% (Fig. 2D), or 5YDFS (*p* = 0.63) 67.5% vs 71.4%, respectively (Fig. 2C), according to long-rank test.

**Table 2:** Demographics divided in MM/ITC positive and negative group

Parameters	MM/ITC positive *N* = 40	MM/ITC negative *N* = 77	*p* value
Sex			
Male, *n* (%)	18 (45.0)	28 (36.4)	0.364*
Female, *n* (%)	22 (55.0)	49 (63.6)	
Age (years)			
Total, mean (SD)	66.7 (12.2)	69.0 (11.1)	0.322**
Male, mean (SD)	68.0 (11.7)	67.7 (11.3)	0.935**
Female, mean (SD)	65.7 (12.8)	69.7 (11.0)	0.185**
Stage			
I, *n* (%)	6 (15.0)	18 (23.4)	0.287*
II, *n* (%)	34 (85.0)	59 (76.6)	
Tumor Differentiation			
Low, *n* (%)	3 (7.5)	6 (7.8)	0.961*
Moderate, *n* (%)	32 (80.0)	60 (77.9)	
High, *n* (%)	5 (12.5)	11 (14.3)	
TNM stage; T			
T1, *n* (%)	0	3 (3.9)	0.337*
T2, *n* (%)	6 (15.0)	15 (19.5)	
T3, *n* (%)	31 (77.5)	57 (74.0)	
T4, *n* (%)	3 (7.5)	2 (2.6)	
Number of D2 lymph *n*odes, mean (SD)	19.3 (8.9)	20.6 (14.0)	0.604**
MM/ITC by Stage			
ITC			
Stage I, *n* (%)	6/6 (100.0)	0/18	
Stage II, *n* (%)	30/34 (88.2)	0/59	
MM			
Stage I, *n* (%)	0/6	0/18	
Stage II, *n* (%)	4/34 (11.8)	0/59	
Long-term survival independent of MM/ITC			
Overall survival, *n* (%)	30 (75.0)	55 (71.4)	0.681*
5-year disease-free survival	27 (67.5)	55 (71.4)	0.660*
Long-term survival independent of MM/ITC by Stage I/II			
Stage I			
Overall survival, *n* (%)	3/6 (50.0)	15/18 (83.3)	0.102*
5-year disease-free survival, *n* (%)	3/6 (50.0)	15/18 (83.3)	0.102*
Stage II			
Overall survival, *n* (%)	27/34 (79.4)	40/59 (67.8)	0.229*
5-year disease-free survival, *n* (%)	24/34 (70.6)	40/59 (67.8)	0.780*
Death due to recurrence, *n* (%)	6 (15.0)	2 (2.6)	0.012*

*MM* micro metastases, *ITC* Isolated tumor cells

*χ^2^ test

**Independent samples *t* test

According to multivariable Cox regression models, adjusting for patient characteristics, there were still no differences in 5YDFS and 5YOS between MM/ITC + and MM/ITC − groups.

However, a significant difference in number of deaths due to recurrence between the MM/ITC groups (*p* = 0.012) (Table 2) was found. Both groups had patients developing liver metastases (5 in MM/ITC + , 2 in MM/ITC −), lung metastases (1 in MM/ITC + , 0 in MM/ITC −), other metastases (2 in MM/ITC + , 0 in MM/ITC −) and local metastases (1 in MM/ITC + , 0 in MM/ITC −) (Table 3).

**Table 3:** Death due to recurrence ML group vs LM group dependent on MM/ITC positive and negative

Patient Id	Age		Sex	T-differ	C-stage	T-size	T-location	MM/ITC	Alive/dead	Metastases	ML/LM
1	67	F		Low	2	4	Cecum	+	Alive	Other	ML
2	48	F		Middle	2	4	Cecum	+	Dead	Liver	ML
3	55	M		High	2	3	Cecum	+	Alive	Liver	ML
4	59	F		Middle	2	4	Ascendens	+	Dead	Lung	ML
5	80	F		Middle	2	3	Cecum	+	Dead	Liver	LM
6	79	M		Middle	2	3	Cecum	+	Dead	Local	LM
7	66	F		Middle	2	3	Cecum	+	Alive	Liver	LM
8	78	M		Middle	1	2	Cecum	+	Dead	Liver	LM
9	44	F		Middle	1	2	Transversum	+	Dead	Other	LM
10	67	F		Middle	2	3	Cecum	−	Dead	Liver	LM
11	77	F		Middle	2	3	Cecum	−	Dead	Liver	LM

*T-differ* tumor differentiation; *C-stage* cancer stage; *T-size* depth of tumor; *T-location* primary tumor location; *MM/ITC* micro metastases/isolated tumor cells; *ML/LM* medial to lateral group/lateral to medial group

## Discussion

The first main finding of this article is that the surgical technique deployed (ML vs LM) has no impact on numbers of MM and/or ITC found in regional lymph nodes in patients with stage I/II right-sided colon cancer. Moreover, the specimen mobilization technique does not influence 5YDFS and/or 5YOS. We did expect that the ML technique would reveal fewer lymph nodes with MM/ITC compared to LM due to central ligation and no-touch of the tumor, and therefore less mobilization of tumor cells during surgery. To our surprise, this could not be demonstrated, in this way implying that the process of lymph node cancer cell spreading is not only a simple consequence of cell shedding due to mechanical shaking, but rather a more advanced process (Chen et al. 2019). In any case it seems that more time is required to achieve dissemination than the time needed to perform a right colectomy. A previous prospective study has compared laparoscopy (medial to lateral) to open (lateral to medial) surgery for colorectal cancer after performing sentinel node biopsy in all patients. They reported significant differences in ITC, while no difference was found for MM (Zaag 2011). This study is characterized by a limited but comparable number of patients (62 laparotomies, 45 laparoscopies) to our study. In contrast to our study, this article had more T3/4 (42) tumors in the laparotomy (lateral to medial) group, while the material consisted of patients with cancer of the colon and rectum. The 5YDFS and/or 5YOS survival data were not provided. The chosen mode of access for the lateral to medial mobilization (laparotomy), as well as patient selection to laparoscopy can also be a confounding factor, while our patients are all operated through open access.

The second most important finding in this article is that the MM/ITC positive patient with stage I/II colon cancer has a significantly higher risk for death from local and/or distant recurrence. Recurrence of the disease is among other factors mostly a function of disease stage and/or surgical technique (Osterman and Glimelius 2018; Tsikitis et al. 2014). While this dataset attempts to neutralize the effect of disease stage on recurrence (only stage I and II disease included) the effect of surgical technique should become more apparent. Since the work of Hohenberger (Hohenberger et al. 2009) published in 2009 a lot of attention has been turned towards preserving an intact Toldt`s fascia to prevent recurrence due to lymph leakage from the mesentery at surgery (Nesgaard et al. 2018). The number of patients that died due to disease recurrence is low and the results obtained need to be interpreted with caution, especially since no difference was found in OS and DFS between the groups. Modern literature contains evidence indicating that both local and distant recurrence are a function of the extent of lymphadenectomy (lymph node harvest) (Hohenberger et al. 2009). Local recurrence rates after right colectomy are reported up to 10% (Augestad et al. 2015; Kishiki et al. 2019). Common sites for local recurrence include the primary tumor position (1.11%) (Augestad et al. 2015), peritoneal carcinomatosis (0.9–1.3%) (Augestad et al. 2015; Kishiki et al. 2019), residual lymph node metastases (0.3–0.8%) (Augestad et al. 2015; Kishiki et al. 2019 Jan) and anastomotic recurrence up to 0.8% (Kishiki et al. 2019). On the other hand, distant metastasis is to the liver (2.7–5.0%) (Augestad et al. 2015; Kishiki et al. 2019) and to the lungs (1–3.9%) (Augestad et al. 2015; Kishiki et al. 2019). All patient recurrences are presented separately in Table 3, the MM/ITC + group with 15.0% death due to recurrence, which is above the values reported in the literature (Augestad et al. 2015; Kishiki et al. 2019), while the MM/ITC- group had 2.6%.

The original article that provided the dataset for the LM group (Faerden et al. 2011) showed a significant difference in 5YDFS between MM/ITC ± groups. LM group dataset contained both left colon and sigmoid cancer patients, which were excluded from our study. The reason for the non-significant 5YDFS in our study can be related to the fact that the above-mentioned study had poorer 5YDFS in the excluded groups. Another explanation can lie in the comparison of only right-sided colon cancer patients in our study. It has recently been recognized that cancer survival in some segments of the colon differ significantly in 5YDFS and 5YOS from other colon segments (Augestad et al. 2015; Zhao et al. 2020).

While analyzing our results it could seem that detecting MM/ITC has the potential benefit of providing both location and extent of the minimal residual disease (MRD) in patients operated for cure of cancer, when compared to liquid biopsy and/or circulating tumor cells (CTC) (Tie et al. 2021; Tarazona et al. 2019). When this statement is in concern the main line of thought is the fact that identifying CTC or liquid biopsy before surgery is of little interest, while the negative result after surgery does not imply MRD. The presence of bone marrow micro metastasis (BMMM) (Viehl et al. 2017) seems to be an indicator of poor survival prognosis at 3 (68.4%) and 5 years (62.7%) follow-up (Murray et al. 2020). In this manner CTC, liquid biopsy cannot be used in the stratification of patients for treatment but rather for follow-up. On the other hand, BMMM and MM/ITC dependent on location have the potential to influence treatment. When considering the correlation between 5YDFS and 5YOS, and numbers of harvested lymph nodes speculation can be made that a proportion of patients have local MRD after surgery. This study shows a higher harvest of lymph nodes in the ML group (26) (Spasojevic et al. 2013; Malik et al. 2020; Nesgaard et al. 2015) in comparison to the LM group (15) while a non-significant, but the nevertheless lower local recurrence rate is noted in the group where a higher lymph node harvest was registered. Most studies have shown MM/ITC detection around 30% in stage I/II, which is the nearly the same proportion as in our study.

This is a retrospective multicenter study on consecutive patients included in two prospective trials within a period of 10 years. The surgery in these two trials had a clear difference in the extent of mesenterectomy performed, which was corrected through the division of the surgical specimen in the ML group. The result of this division was still a highly significant difference in the numbers of harvested lymph nodes between the respective level of dissection 1/2 and 3 volumes. This can be explained through the line of division between the respective areas of dissection in the ML group (through a line 10 mm towards the right of the superior mesenteric vein beginning at 10 mm caudal to the ileocolic artery origin and ending at 5 mm cranial to the middle colic artery origin), while the distance of the medial edge of the mesentery to the SMV was unknown in the LM group. The same pathologist (SNA) and pathology lab performed all the analyses except for six patients that were analyzed by US, using the same technique rendering the results comparable. One of the drawbacks of this study is the fact that the data used for comparison were collected from two trials, in two different periods, to secure open surgical access for both groups, as well as access to the same pathology lab.

## Conclusion

There are no differences in MM/ITC numbers, as well as 5YOS and 5YDFS, when comparing medial to lateral and lateral to medial specimen mobilization when operating right-sided colon cancer. Presence or absence of MM/ITC does not affect 5YOS and 5YDFS but can be a potential prognostic factor for death due to recurrence.

## Data Availability

Available on request.
